# A Soft Tissue-Borne Patient-Specific Guide for Foreign Body Removal in the Facial Region

**DOI:** 10.7759/cureus.73698

**Published:** 2024-11-14

**Authors:** Amjad Shhadeh, Shadi Daoud, Adeeb Zoabi, Samer Srouji, Fares Kablan

**Affiliations:** 1 Department of Oral and Maxillofacial Surgery, Galilee College of Dental Sciences, Galilee Medical Center, Nahariya, ISR; 2 The Azrieli Faculty of Medicine, Bar-Ilan University, Safed, ISR

**Keywords:** foreign bodies, minimally invasive surgery, patient-specific guide, soft tissue-borne guide, virtual surgical planning

## Abstract

Foreign body removal in the facial region poses significant challenges due to the complex anatomy and the proximity to critical structures. This study introduces a soft tissue-borne patient-specific guide (PSG) designed to enhance precision and minimize invasiveness in foreign body removal. Four patients underwent foreign body removal using PSGs, with CT and cone-beam computed tomography (CBCT) imaging employed for segmentation and detailed analysis of both hard and soft tissues. This approach enabled the planning and design of stable and personalized guides. Ultrasound tracing was used for intraoperative verification. All procedures were successful, with minimal scarring, no complications, and reduced operative time. The use of PSGs improved surgical accuracy and efficiency, highlighting their potential for foreign body removal in other regions of the body and for broader clinical applications in maxillofacial surgeries.

## Introduction

Foreign bodies embedded in soft tissues are generally harmless, but complications such as chronic pain, inflammation, infection, granuloma formation, functional impairment, vascular compression, or neurological symptoms can necessitate their removal [1]. Additionally, metallic foreign bodies may necessitate removal when they contraindicate magnetic resonance imaging (MRI) [2]. The removal process becomes particularly challenging in delicate areas like the facial region due to limited access and the proximity of vital structures. Accurately locating and extracting these foreign bodies without causing additional trauma to surrounding tissues is a complex and delicate task [3].

Whenever possible, foreign bodies should be removed during the initial phase of wound management. These objects, which vary in shape, size, and material nature such as wood, glass, or metal, often demand different imaging modalities for accurate identification [4]. The choice of imaging technique, whether plain radiography, computed tomography (CT), MRI, or ultrasound, is based on the location and material of the foreign body [5,6].

Over time, various innovative techniques have been developed to address these challenges, including needle-guided techniques, magnet-assisted removal methods, and image-guided surgical navigation approaches [1,7-10]. While patient-specific implants (PSIs) have demonstrated high accuracy in bone-borne applications, their effectiveness in soft tissue-borne scenarios is considered less consistent due to the dynamic nature of soft tissues, which can shift and lead to inaccuracies.

To overcome the limitations of soft tissue-borne applications, we propose a novel approach for designing a patient-specific guide (PSG) tailored to the facial region. This method integrates advanced imaging modalities alongside a detailed analysis of both hard and soft tissues. Key factors such as tissue elasticity, Hounsfield units (HU), and soft tissue thickness, along with facial morphology, are carefully evaluated to enhance guide stability. The combination of these analyses ensures greater precision during PSI design. Additionally, we utilize intraoperative ultrasound guidance to improve the accuracy of foreign body localization and removal.

By integrating this technique, our approach enhances the precision and safety of minimally invasive procedures. The use of PSG represents a valuable advancement in maxillofacial surgery and offers potential for foreign body removal in other regions of the body, offering a versatile solution across various anatomical areas.

## Materials and methods

This study reports on four patients who underwent metallic foreign body removal from the facial region using soft tissue-borne PSGs. The guides were designed, planned, and fabricated in-house at the Department of Oral and Maxillofacial Surgery, Galilee Medical Center, Nahariya, Israel, between February 2023 and March 2024.

Patients were eligible for inclusion if they had metallic foreign bodies embedded in the facial soft tissues and were candidates for removal using a PSG based on preoperative imaging and planning. Exclusion criteria were applied to patients with avulsion wounds or foreign bodies deeply embedded in a manner that required extensive surgical exposure beyond the scope of minimally invasive techniques.

Foreign bodies and facial soft tissue segmentation

**Figure 1:**
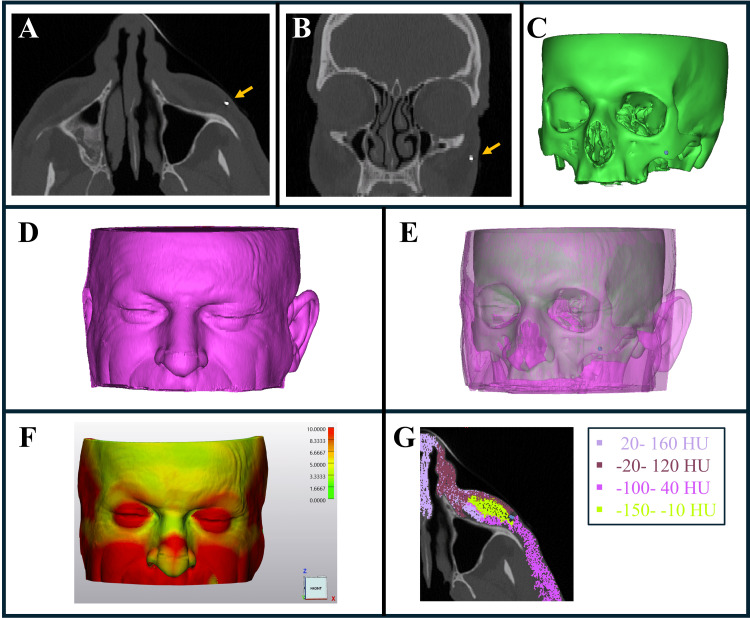
Detailed segmentation and analysis of foreign body and adjacent soft tissues in the facial region. (A) Axial CBCT slice indicating the metallic foreign body (yellow marker) in the left cheek area. (B) Coronal slice illustrating the foreign body embedded in the subcutaneous tissues. (C) Segmentation of the bone (green) and foreign body (blue), extracted from the CBCT scan. (D) Segmentation of the facial soft tissue (purple). (E) Combined segmentation of bone, soft tissue, and foreign body with a transparent overlay of the soft tissue. (F) Soft tissue thickness assessment displayed as a heat map, revealing that the periorbital and nasal region is the thinnest and has the most bony support. (G) Hounsfield Unit (HU) evaluation of the adjacent soft tissue, highlighting the more elastic tissue on the medial side of the foreign body. CBCT: cone-beam computed tomography The images belong to Case 1.

The process begins with high-resolution imaging using CBCT (Planmeca Viso G7, Planmeca, Helsinki, Finland) or CT (Siemens Somatom X.cite, Siemens Healthineers, Forchheim, Germany) with a 1 mm slice thickness. This is followed by the segmentation of both foreign bodies and the surrounding bony and soft tissues using Mimics software (Materialise, Leuven, Belgium) (Figures 1C-1E). Segmentation is performed by applying thresholding techniques, with soft tissue segmentation based on its HU range (typically -100 to 100 HU) and bone segmentation at higher HU ranges. Foreign body segmentation is typically determined by its material type, with metallic objects generally exceeding 2000 HU, allowing for accurate differentiation from surrounding tissues.

Soft tissue evaluation

A key component of this method is the evaluation of soft tissues, which is conducted through several crucial steps. After identifying the region of interest, the surrounding area is analyzed for soft tissue thickness using heat map evaluations (Figure 1F), as well as for bony support and overall facial morphology. Additionally, the HU range of the soft tissues is assessed as an indicator of tissue elasticity (Figure 1G), providing critical insights into how the tissue is likely to behave during surgery. Known zones of tissue elasticity in the face are also taken into account to ensure a more precise and customized approach to guide design [11,12].

Guide design

**Figure 2:**
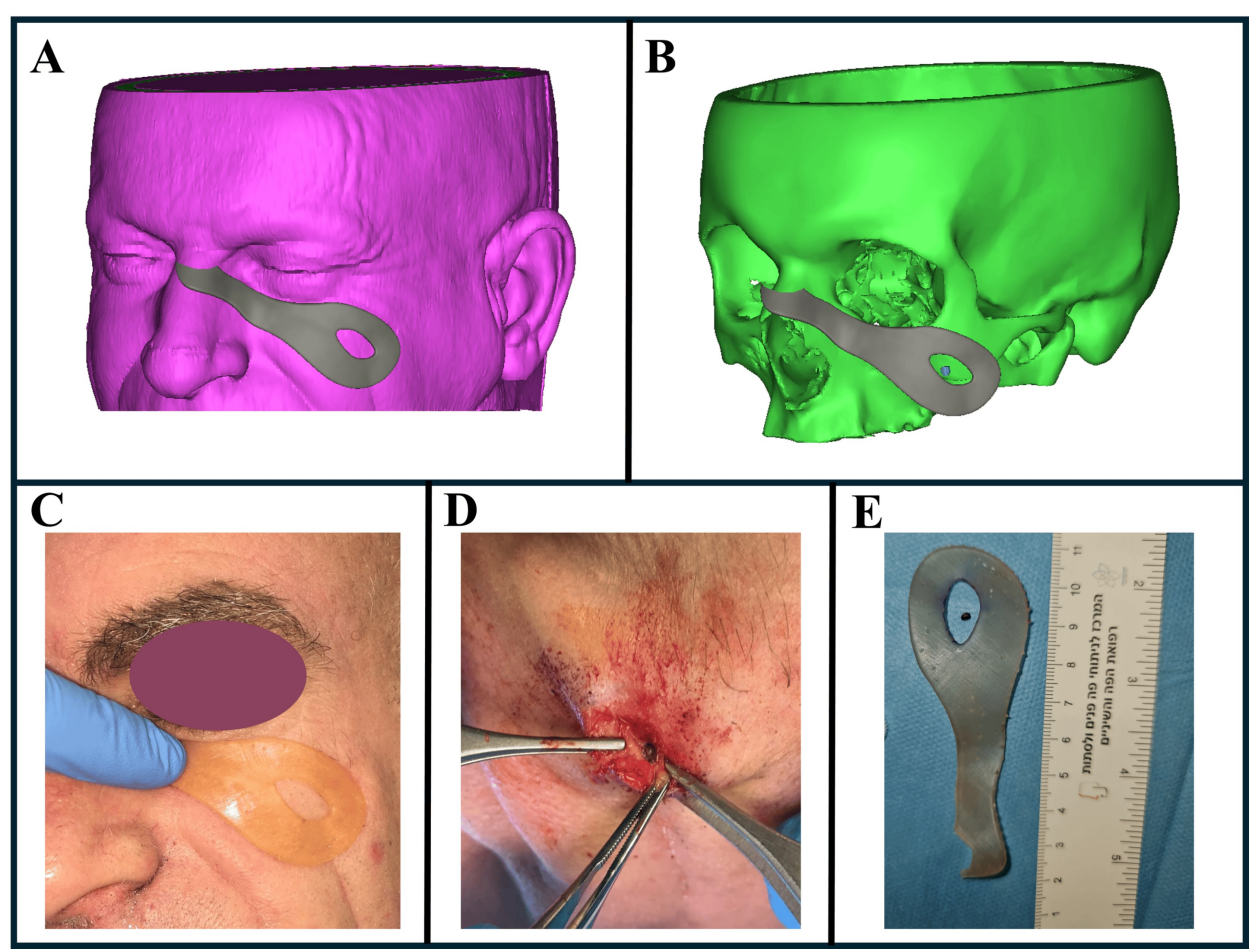
Guided foreign body removal using soft tissue-borne patient-specific guide. (A) The designed guide was positioned on the facial soft tissue, incorporating key soft tissue features and anatomical morphology. (B) Guide visualization without the facial soft tissue, highlighting the location of the foreign body (blue). (C) Intraoperative application of the guide, demonstrating a positive fit on the patient’s facial soft tissue. (D) Minimally invasive removal of the foreign body using the guide. (E) Postoperative view of the patient-specific printed guide and the extracted foreign body. The images belong to Case 1.

The PSG is designed based on segmentation data and the evaluation of soft tissues, with particular attention to areas with less tissue elasticity, greater bony support, and regions with thinner soft tissue or reduced soft tissue mobility. The guide is meticulously tailored to the patient’s unique anatomy, using natural undercuts to ensure a precise fit over the soft tissue in the target area (Figures 2A, 2B). The guide was designed with an opening centered on the foreign body's location, with additional space incorporated to accommodate the surgical approach. This design allows the surgeon to accurately localize the foreign body, facilitating a minimally invasive procedure. The guide is fabricated using 3D printing technology, specifically utilizing the biocompatible MED610 transparent resin with the J5 MediJet 3D printer (Stratasys, Minnesota, United States).

Surgical procedure

The procedure begins with sterilization of the facial region, followed by the placement of the PSG over the predefined area. Local anesthesia is administered, and an incision is made either along an existing scar or at the central location indicated by the guide. Using the PSG, the surgeon carefully extracts the foreign body through a minimally invasive approach. Intraoperative ultrasound was used throughout the procedure to confirm the precise location of the foreign body, ensuring accurate removal and reducing the risk of complications. This combination of preoperative planning, customized guides, and real-time imaging enhances the safety and efficiency of the procedure.

## Results

**Figure 3:**
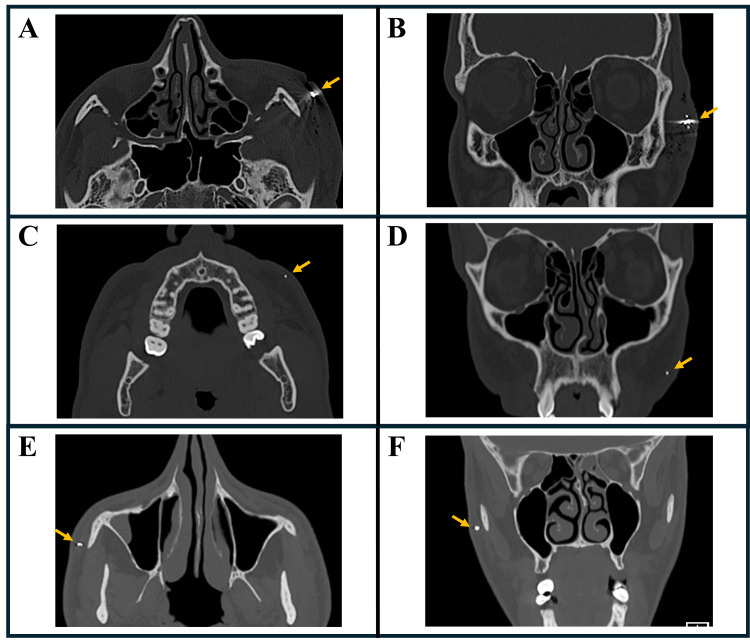
CT imaging of metallic foreign bodies in cases 2-4. CT images showing the precise locations of metallic foreign bodies (yellow arrows) in the facial regions of cases 2-4. (A, B) Case 2: Axial and coronal slices reveal three metallic fragments in the left zygomatic area. (C, D) Case 3: Axial and coronal views display a single metallic fragment in the left buccal region. (E, F) Case 4: Axial and coronal sections highlight a metallic fragment in the right zygomatic area.

In our study, four patients underwent foreign body removal from the facial region using soft tissue-borne PSGs. The first patient was a 47-year-old male with a 2 mm metallic fragment embedded in the subcutaneous tissue along the left border between the infraorbital and zygomatic regions, resulting from an explosive injury. Although asymptomatic, the fragment removal was necessary to enable an MRI for evaluating hearing loss. Figures 1-2 illustrate the diagnostic imaging, guide design, and treatment process for this case. The second patient, a 23-year-old male, had sustained a gunshot injury, leaving three metallic fragments embedded in the subcutaneous tissue of the left zygomatic region: a 6 mm fragment and two 2 mm fragments in the subcutaneous tissue in the left zygomatic area, which caused chronic localized pain (Figures 3A, 3B). The third case involved a 30-year-old male with a 2 mm metallic fragment embedded in the subcutaneous tissue of the left buccal region, also from an explosive injury, causing persistent discomfort and chronic localized pain (Figures 3C, 3D). The fourth patient, a 22-year-old male, presented with a 3 mm metallic fragment embedded in the subcutaneous tissue of the right zygomatic region following an explosive injury, resulting in chronic localized pain (Figures 3E, 3F). The duration from injury to foreign body removal for each patient is indicated in Table 1, which summarizes the patients' demographic and clinical characteristics.

**Table 1:** Patients and foreign bodies removed using soft tissue-borne patient-specific guide. M: male; yr: year; mo: month

Case	Patient Age (yr)/Gender	Material	Quantity	Site	Duration (mo)	Etiology
1	47/M	Metal	1	Border between infraorbital and zygomatic area	26	Explosive injury
2	23/M	Metal	3	Zygomatic area	13	Gunshot injury
3	30/M	Metal	1	Buccal area	4	Explosive injury
4	22/M	Metal	1	Zygomatic area	1.5	Explosive injury

The use of PSGs enabled precise localization and minimally invasive extraction in each case. The mean operative time following guide placement was approximately 15 minutes. No intraoperative or postoperative complications occurred, and neurological as well as functional assessments conducted before and after surgery showed no sensory or motor impairments.

Cosmetically, there was no noticeable scarring at the surgical sites. At the six-month follow-up, all patients reported complete resolution of symptoms, including relief from chronic pain and functional limitations. Aesthetic outcomes were highly favorable, with full restoration of facial form and function.

The use of PSGs improved procedural efficiency, allowing for accurate foreign body removal while minimizing trauma to surrounding tissues. This approach demonstrated enhanced surgical precision, faster recovery, and excellent patient outcomes, highlighting its potential for broader applications in maxillofacial surgery and beyond.

## Discussion

Removing foreign bodies from the soft tissues of the facial region poses significant challenges due to the complex anatomy and the proximity of vital structures. Current methods for foreign body removal, such as magnet-assisted, needle-guided, and image-guided navigation systems, each offer specific benefits but also come with limitations. For example, magnet-assisted techniques, while fast and inexpensive, are limited to superficial metallic objects and are less effective when the foreign body has been present for an extended period [1]. Similarly, needle-guided approaches are simple and cost-effective but are less suitable for foreign bodies in deeper or high-risk areas [9]. Furthermore, precise identification of the foreign body through clinical examination is essential for the successful application of this technique [9]. Image-guided navigation systems provide higher accuracy, especially in complex cases, but are hindered by high costs, a steep learning curve, and a time-consuming setup [7,13,14].

The introduction of soft tissue-borne PSGs has the potential to address the challenges of foreign body removal by offering an accurate and minimally invasive solution. Advanced imaging techniques, such as CT and CBCT, combined with segmentation tools, enable precise localization of the foreign body and customization of the guide to the patient's unique soft tissue and skeletal anatomy. By designing these guides, we addressed the key challenge of the inherent mobility of soft tissues, which can lead to inaccuracies. While some movement remains, our approach minimizes its impact, ensuring more stable guide placement during surgery and enhancing the surgeon’s ability to perform targeted removal with minimal collateral damage. As a result, in this study, all patients underwent successful foreign body removal with minimal scarring and no postoperative complications, and the mean operative time was reduced.

A key advantage of PSGs is their ability to preserve the delicate balance between functional and cosmetic outcomes, particularly in the facial region where even minor incisions can result in noticeable scarring. By guiding the surgeon with precision, PSGs minimize the need for extensive dissection, thus preserving both the form and function of the affected area. Ultrasound plays a critical role in real-time localization of the foreign body during surgery, enhancing procedural accuracy and supporting precise foreign body extraction with minimal risk of intraoperative errors. Looking ahead, augmented reality (AR) holds potential as a supplementary tool by providing real-time visualization to assist in locating or confirming the PSG’s position. As AR technology advances, it could further improve surgical precision and efficiency, offering a promising future in maxillofacial procedures [15].

Despite the promising results, the limitations of this study, including the small sample size, must be acknowledged. Future research with larger cohorts is necessary to validate these findings and explore further refinements to PSG design, such as incorporating guiding sleeves for surgical instruments. Additionally, this technique shows potential for foreign body removal in other areas of the body and could be adapted for use in procedures like soft tissue biopsies and injections, making it a valuable subject for future research.

## Conclusions

In conclusion, while the design and preparation of soft tissue-borne PSGs require additional time and resources, our approach to designing these guides has demonstrated clear advantages. These guides enhance the precision of foreign body localization and minimize surgical trauma, offering both functional and cosmetic benefits. They allow for more accurate, minimally invasive procedures, making them particularly useful in complex regions like the face. Moreover, this technique holds potential for foreign body removal in other areas of the body and could be adapted for additional procedures, such as soft tissue biopsies and injections. As a result, these guides offer promising potential for broader clinical application, improving patient outcomes and setting a new standard in surgical precision.
